# Understanding the situation of bystanders to inform anti-bullying interventions

**DOI:** 10.3389/fpsyg.2024.1116860

**Published:** 2024-10-14

**Authors:** Samantha K. Cohane, Barry H. Schneider

**Affiliations:** Department of Psychology and Neuroscience, Boston College, Chestnut Hill, MA, United States

**Keywords:** bullying, victimization, bystanders, prevention, aggressive behavior

## Abstract

Bystanders who witness a bully-victim exchange at their school differ from bystanders who witness many incidents of violence in their community, due to the web of mutual relationships that exist within a school setting. Research conducted in many countries has revealed a variety of ways in which peers too often support and encourage bullies, thereby reinforcing the bully’s behavior and further marginalizing their victims. This elucidates the potential benefits of channeling bystanders’ neutrality into opposition to bullying that is beneficial and supportive of victims. This goal has been incorporated into preventive anti-bullying interventions that have been implemented and evaluated. In this paper, we focus on the contention that systematic anti-bullying intervention in schools and communities can affect the stance of bystanders. We pay particular attention to the KiVa program, which was originally developed in Finland but has since been implemented in schools throughout many countries due to promising data regarding its effectiveness as well as its emphasis on the role of bystanders. We discuss the data documenting the effectiveness of preventive school-based anti-bullying programs, considering the proposition that these programs reduce bullying at least partially by improving bystander behavior. Despite ample evidence that KiVa reduces bullying, there is little specific data showing that the effects of KiVa are mediated by improvements in bystander behavior. The paper concludes with speculation about the possibility of a more direct and active mobilization of student mediators and student leaders to influence the behavior of bystanders in their classrooms and schools.

## 1 Introduction

Public concern about inaction by bystanders who witness an act of violence was aroused by the murder of Kitty Genovese in New York City at 3 a.m. on a cold March night in 1964. Genovese was attacked in full view of neighbors who shared the courtyard of her apartment building. None of them called the police during the 35-min attack until it was over. The bystander effect shocked the U.S. and the world so strongly that the case is still discussed in scholarly writings (Kassin, 2017). The incident also inspired considerable theory-building and research in psychology. Casey et al. (2017) summarize the main elements of the *situational model of bystander behavior* espoused by Darley and Latané (1968). First, bystanders must notice the behavior in question and must perceive it as problematic, serious, and wrong. They must recognize that they have an obligation to intervene in the situation. Finally, they must know what to do or how to decide what to do. Editorials in newspapers around the world decried the anonymity of urban life, which seems to render people indifferent to human beings they do not know.

There is relatively little mention of the fact that neighbors may have failed to help Ms. Genovese because she was an individual of minority sexual orientation (Kassin, 2017), as are many victims of bullying in schools today. There has been very limited research on the role of bystanders in incidents of homophobic bullying at school. An important exception is a study by António et al. (2023), who found that contact with a hypothetical fellow pupil of minority sexual orientation increased the likelihood of younger, female participants, but not others, to intervene on the victim’s behalf. Unfortunately, the major published anti-bullying programs, i.e., KiVa, Second Step and the Olweus program, do not focus specifically on homophobic bullying. It has been found, however, that by insisting that all bullying is wrong regardless of the victim’s personal characteristics, they do reduce the victimization of students of minority sexual orientation, as by the gold-standard KiVa program (Granero Andujar and Manzano-Leon, 2018). Preliminary results of targeted interventions designed to reduce homophobia, however, suggest that further gain, including more helpful bystander behavior in cases of homophobic bullying, might result if more specific content on homophobia were included (Amadori et al., 2023).

Only one in five or six pupils are typically identified by their classmates as bystanders who would intervene actively to defend the victim if they witnessed a fellow pupil being bullied at school. Other bystanders would do nothing, or, even worse, assist the bully or approve of their bullying (Salmivalli, 2010), even though many of them express the intention to help the victim even though they do not follow through and act on their intentions (Boulton et al., 2002). Scholars recognize that only some parts of the Darley and Latané model of bystander behavior apply to decision-making concerning bully-victim encounters at school. Those encounters are not usually anonymous as the bully and victim are pupils at the same school. Bystanders may, however, remain uninvolved because they believe that someone else is likely to intervene.

In their pioneering work on bystander behavior in bully-victim encounters at school, Salmivalli (e.g., 2010) identified other aspects of decision-making regarding whether to intervene as a bystander to bullying at school, introducing several constructs not considered in the classic research on the bystander effect. The most important issue mentioned is the need and desire of bystanders regarding their own popularity among their school peers: Bullies, their assistants and those who support and encourage them may feel that their behavior will make them more popular, which tends to be particularly important to them. In this instance, popularity may mean that peers consider them powerful and admire their strength, not necessarily that they are liked or sought after as friends. It has indeed been found that bullying or helping the bully can make an individual popular in some schools and classrooms but not all (Sentse et al., 2007).

Working with a large sample of Italian adolescents, Pozzoli et al. (2012) confirmed that some of the elements in Darley and Latané’s classic model do apply to thinking about intervening during bullying incidents at school, at least according to the self-reports of the potential intervenors. Their research also elucidated several other important variables that play a role in adolescents’ decisions about whether to intervene when they witness a bullying episode. Pozzoli and Gini found that the participants’ decision to defend the victim depended upon their own attitudes regarding bullying and victimization as well as the feeling that they had a moral responsibility to intervene. In addition to variables suggested by Darley and Latané, pressure by parents and peers emerged in the Italian data as potent predictors of adolescents’ decision-making. Once more, the methodology used in this study may have affected the findings. Although self-reports were probably the only viable way of obtaining data on decision-making from a large sample, self-reports have various known biases, including the tendency to project a socially desirable image. The self-results of aggressive children are particularly questionable (e.g., Huizinga, 1991). Self-reports have the advantage, however, of providing the best access to mental processes accruing within the mental space of an individual.

Many personal characteristics of bullies, victims, and bystanders have been studied. Among these, empathy is highly relevant to the processes that lead bystanders to elect to act in a prosocial way and come to the aid of a victimized fellow student. Specifically, the differences between the two subtypes of empathy, cognitive empathy and affective empathy, as well as the subsequent influence both exert on bystander behaviors have been examined.

Researchers have devoted considerable attention to the study of the associations between empathy and bystander behavior. A three-level meta-analysis of 35 independent studies, by Deng et al. (2021), sought to address the discrepancy in the research regarding empathy’s impact on bystander behavior. While many studies report a strong correlation between empathy and bystander defending, others failed to reveal a statistically significant relationship between the two. Deng and colleagues also analyzed whether the type of empathy moderates its effect on bystander behavior. The meta-analysis revealed that there is indeed a positive correlation between empathy and defending behavior from bystanders some interventions designed to improve empathy attempt to get participants to empathize with real people in their lives; others involve hypothetical protagonists, as in stories. Scott et al. (2022) designed an intervention to promote positive bystander behavior using children’s literature. Empathy was an important component of their intervention with elementary school students from low-SES families. The results indicated an improvement in bystander behavior for the experimental group but not the control participants.

## 2 Method

With evidence that bystanders can improve situations of bullying, it is important to evaluate the effectiveness of potential interventions that specifically target bystanders. We do so by compiling a detailed narrative review of a selection of studies examining KiVa, the most promising of the anti-bullying interventions that specifically target bystander behavior. We examined a variety of randomized control trials (RCTs) of the KiVa program, which took place across multiple different countries, by looking at KiVa’s ability to reduce both bullying and victimization. Our review involved careful scrutiny of these studies in order to discover whether the outcome data might be influenced by the sampling, intervention method, research design, and outcome measure selected by the authors. All studies examined include outcome measures related to both bullying and victimization. In our review, we separate studies conducted by the researchers who developed KiVa from studies implemented by other researchers outside of Finland (where the program was most developed). We believe that this generalizability is a fundamental part of the general appraisal of KiVa. We hypothesized that KiVa would be effective at reducing rates of both bullying and victimization, given the growing body of research underscoring the importance of the role bystanders play in bullying situations.

### 2.1 KiVa: a prevention program that highlights bystander intervention

KiVa, an Antibullying Program developed at the University of Turku, Finland, specifically focuses on and targets bystander behavior. KiVa asserts that its cornerstones are prevention, intervention, and monitoring (*What is Kiva?*).^1^ The other major school-based prevention programs, Second Step and the Olweus program, provide only general instructions to pupils about not supporting the bully, reporting bullying incidents to an adult, etc. Bystander behavior is featured prominently in a much briefer intervention for secondary-school students called *Say Something* (Booker et al., 2023). This intervention consists only of a single 150-min workshop. Despite the small scale of the intervention, the intervention participants reported intervening to support victims of bullying more frequently than members of the control group. Further study and, especially, follow-up data are needed.

KiVa equips teachers with a variety of resources including training modules, materials, and lesson plan manuals. It also includes resources for parents such as a guide that includes general information about bullying, bullying detection, and bullying prevention. According to its website, KiVa is the most rigorously studied antibullying program. The creators of KiVa hypothesized that the less positive reinforcement a bully receives from his or her classmates, the less rewarding bullying will be, which will decrease the motivation to bully to begin with (Kärnä et al., 2011b). Indeed, KiVa assumes that bullying is a social, group phenomenon, (Salmivalli et al., 1996) meaning that bystander behavior modulates bullying (Kärnä et al., 2011a). For this reason, KiVa puts a particular emphasis on shaping the attitudes and behaviors of students who are bystanders.

The bystander material in KiVa does address some components of the situational model of bystander behavior that has emerged from broader research on the bystander effect, introduced earlier. In KiVa, potential bystanders learn very clearly that bullying is a serious problem. They also learn what they can and should do if they witness bullying at school. The identification of bystanders who have fulfilled their responsibilities reduced the anonymity that has been found to lead to standing by idly. Besides being the antibullying program that focuses most specifically on bystander behavior, it is also the one bolstered by the greatest among evaluation data.

The program consists of three separate versions, each tailored to a specific age group (Grades 1–3 (ages 6–9), Grades 4–6 (ages 10–12), and Grades 7–9 (ages 13–16)). In all three versions of the program, KiVa is divided into two types of actions: universal actions and indicated actions. Universal actions are targeted to the entire school and are meant to be implemented, regardless of the circumstances. These actions include 20 h of classroom instruction time, consisting of 10 lessons that cover a variety of topics including peer pressure, respect, and the negative outcomes of bullying. The objective of these lessons is to increase students’ empathy toward bullying victims, spread awareness about the issue of bullying as well as the role the “group” plays in addressing and preventing it, and equip kids with adequate skills to intervene in situations of bullying, thereby increasing their self-efficacy. Students can strengthen their knowledge of these subjects and improve their defending skills by participating in a computer game as part of KiVa (Poskiparta et al., 2012). The game is broken into three parts: “I KNOW,” “I CAN,” and “I DO.” The “I CAN” and “I DO.” Parts of the game specifically target bystander behavior by teaching students how to do the right thing in situations of bullying and motivating them to defend victims (“I CAN”) and then giving them real-world scenarios where they can put these new defending skills into practice (“I KNOW”). Indicated actions are only utilized in instances when bullying is reported to the school. A team consisting of three teachers (or other school faculty) and the bullied student’s classroom teacher is created to address the specific case of bullying. The classroom teacher holds a meeting with between two and four students who exhibit prosocial behaviors and who are considered to be of high social status. Currently, there is no standardized method of picking these students, so it may be beneficial to systematize thus in future evaluation research and/or future revisions revision of the KiVa program. Individual and small group discussions with victims and bullies are implemented, complemented by follow-up meetings.

### 2.2 The effectiveness of KiVa in Finland

The studies by Salmivalli and her Finnish colleagues that document the effectiveness of KiVa are many, including several featuring longitudinal follow-up and control conditions. The very first RCT of KiVa took place during the 2007–2008 academic year when KiVa was implemented in schools throughout Finland in grades 4 through 6 whilst being studied for effectiveness (Kärnä et al., 2011a). 78 schools participated in the study, 39 of which were in the KiVa condition (4,207 students) and the other half of which were in the control condition (4,030 students). The study established KiVa as an effective program to target bullying and demonstrated that after only 9 months of implementation, KiVa was effective at reducing both victimization and bullying. In addition to demonstrating the overall effectiveness of KiVa, Kärnä et al. (2011a) highlighted KiVa’s potential to increase prosocial bystander attitudes and empathy, as well as improve bystander behavior. At Wave 2, students in the intervention condition showed increased anti-bullying attitudes and increased empathy. Unfortunately, these effects were rendered null by Wave 3. A similar trend was seen when bystander behavior was analyzed. At Wave 2, students in the intervention condition exhibited defending behaviors more frequently than their counterparts in the control condition. Yet, once again the effect of the intervention on defending behavior was not significant by Wave 3. Despite this, there was a decrease in assisting and reinforcing behaviors at Wave 3. These results are indicative of KiVa’s effectiveness at improving bystander responses to bullying, and the areas where KiVa was not effective (i.e., significant effects at Wave 2 that were nonsignificant at Wave 3) guide future research. Future studies should investigate why might this change be occurring, and how KiVa can be improved to create a long-lasting impact on bystander behavior.

The goal of another study by Saarento et al. (2015) was to uncover which mechanisms in KiVa lead to this reduction of reported victimization amongst students in grades 4 through 6 who have undergone the program. The results confirmed that students in KiVa schools perceived a significant increase in the number of classmates as defenders of the victim and an increase in their classmates’ defending behavior. In contrast to their hypothesis, however, these perceptions were not predictive of future bullying. Participants in the KiVa program showed an increase in affective empathy as well as in antibullying attitudes (Saarento et al., 2015). Results of the study revealed that antibullying attitudes acted as a mediator for self-reported bullying measures, though this was not the case for affective empathy. As well, participants also perceived an increase in their teacher’s overall disapproval of bullying, which led to a statistically significant reduction in self-reported bullying. Results showed that the teacher’s attitudes toward bullying also mediated KiVa’s effects.

Garandeau et al. (2021) similarly considered KiVa’s impact on empathy, querying whether the KiVa program increases cognitive and/or affective empathy in its participants. Further, they questioned if such effects were consistent across all populations (i.e., people of different genders, races, etc.). They discovered that KiVa had a positive impact on affective empathy, and this effect was seen consistently across students of all demographic backgrounds. At the individual level, affective empathy was significantly higher for participants in the KiVa program, though the effect size was quite small. At the classroom level, a significant positive effect of KiVa on affective empathy was also seen and had a slightly larger, but nonetheless small effect size. The same positive effects were not seen on cognitive empathy. While Garandeau et al. (2021) did not measure KiVa’s direct influence on bystander behavior, the increase in affective empathy after participation in KiVa might act as a mediator variable and lead to an increase in defending behaviors, considering previous research that demonstrates a positive correlation between bystander defending and affective empathy (Deng et al., 2021).

Garandeau et al. (2023) sought to determine the degree of impact KiVa has on defending behaviors, as well as the individual-level factors that influenced such defending behaviors as measured by the Participant Role Questionnaire (Salmivalli and Voeten, 2004). The results showed that at 5 months and 9 months, KiVa had a positive effect on defending behavior. While the result was not significant at 9 months when controlling for baseline and 5 months at the same time, there was still a significant indirect effect of KiVa on defending behaviors at 9 months via defending at 5 months (Garandeau et al., 2023). The only significant mediator found to have a consistently positive effect on defending behaviors was responsibility to intervene, when measured at 5 months.

Despite these positive results, one study raises possible concerns regarding KiVa’s effectiveness on bystander behavior. The results of Kärnä et al. (2013), an RCT that studied KiVa’s effectiveness on a population of students in Grades 1–3 and Grades 7–9 (populations that, at the time of the study, had yet to be studied for KiVa’s effectiveness) showed that those in the intervention condition exhibited a decrease in defending behaviors after undergoing KiVa. All students were also asked to nominate an unlimited number of peers as defenders. Students were able to simply report “no one,” if they could not identify a peer whom they saw as a defender. While the intervention effect of KiVa was statistically significant, it was not in the hypothesized or desired direction: defending actually *decreased* in the intervention condition. This was consistent across all age groups in the study, though the effect increased with age. Future research should seek to address these differing results between studies.

### 2.3 The effectiveness of KiVa outside of Finland

KiVa has also proven to be effective outside of its country of origin. In a Dutch trial of KiVa, a support-group approach was added to the indicated actions protocol, and its subsequent impact on victimization and defending was measured (Van Der Ploeg et al., 2016). To measure any change in defending bystander behavior, data was collected from students in October and again in May. Students who indicated that they were victims of bullying were asked to nominate defenders. Change was indicated by measuring the difference between the total number of defenders each victimized student nominated in October and May. To analyze the differences between students who participated in the support group approach and those who did not, students in both conditions were statistically matched to one another. Victims with a support group had more defenders by May than those who did not have a support group. Descriptive statistics also convey these results, with 52.6% of victims in the support group condition reporting an increase in defending as opposed to only 34.7% of victims in the control condition reporting an increase in defending. The results of Van Der Ploeg et al. (2016) show that a support-group approach to increasing bystander defending is effective.

In further support of KiVa’s effectiveness at improving bystander behavior in countries other than just Finland, data from a Chilean trial of KiVa revealed improvement in the psychosocial adjustment of youths enrolled in schools that offered the KiVa program (Valenzuela et al., 2022). Psychosocial adjustment was measured by a self-report which included questionnaire items related to prosocial behavior, which probably includes helpful bystander behavior although bystander helping was not specifically isolated. Further analysis of this data could allow the researchers to pinpoint if the prosocial behavior was a main component impacting the improvement in psychosocial adjustment, or if it may have been another factor.

The following table summarizes the eight major trials of KiVa that have yielded results and taken place outside of its country of origin, Finland, during the period covered by our literature review. The inclusion criteria for our literature review were that the study had to include, first of all, quantitative data on the effects of Kiva. It had to either be a published study indexed in PSYCINFO before DATE or a study mentioned in the articles already retrieved; and the trial of KiVa had to be conducted outside of Finland (Table 1). Eight studies emerged, including Nocentini and Menesini (2016), Van der Ploeg et al. (2016), Swift et al. (2017), Green et al. (2020), Axford et al. (2020), Huitsing et al. (2020), López-Catalán et al. (2022), and Valenzuela et al. (2022).

**Table 1:** Results of major evaluation studies of Kiva outside of Finland: bullying perpetration and victimization.

Source	Country	Grades (ages)	Condition(s)	Sample size	Data collection points	Bullying measures	Bullying results	Victimization measures	Victimization results
Nocentini and Menesini (2016)	Italy	4, 6 (mean = 8.85, 10.93)	Intervention (KiVa) Non-treatment Control (No KiVa)	1,039 —Intervention 1,003 —Control	Pre-test (beginning of school year) Post-test (end of school year)	Self-reports: Florence Bullying and Victimization Scale (Palladino et al., 2016) Global key question—two items from Olweus Bully / Victim Questionnaire (Olweus, 1996)	Bullying significantly decreased over time in the experimental group, not in the control group (stronger effect in Grade 4)	Self-reports: Florence Bullying and Victimization Scale (Palladino et al., 2016) Global key question—two items from Olweus Bully / Victim Questionnaire (Olweus, 1996)	Odds of being a victim were significantly lower for students at KiVa schools. Victimization significantly decreased over time in the experimental group, not in the control group (stronger effect in Grade 4)
Van der Ploeg et al. (2016)	The Netherlands	2–6 (7–12)	KiVa KiVa + support group Control (“care as usual”)	38 schools — KiVa 28 schools— KiVa + support group	Pre-assessment (May 2012) October 2012, 2013 May 2013, 2014	Self-report: Online questionnaire (Kärnä et al., 2011b) Peer reports: Peer nominations of both bullies and defenders (as previously used in research)	Victims with a support group had more defenders by the end of the school year than did those without a support group (*z* = −2.39, *p* = 0.01) yielding an effect size of 0.31.	Self-report: Online questionnaire (Kärnä et al., 2011b); Olweus Bully / Victim Questionnaire (Olweus, 1996); Peer reports: Peer nominations of victims (as previously used in research)	For the two intervention conditions there was no difference in victimization (z = −0.03, *p* = 0.49) but those with a support group were less frequently victimized (z = −3.27, *p* = 0.00). In the short term, victimization improved, but these changes were not sustained leading to no difference being found at the end of the school year for the two intervention conditions.
Swift et al. (2017)	United States	4–5	Pre-KiVa Post-KiVa (same group, 1 year later)	1,409	Pre-test (beginning of school year) Post-test (end of school year)	Self-reports: Bullying Behavior Scale “BBS” (Austin and Joseph, 1996) Peer-reports: BBS (Austin and Joseph, 1996) with peer nominations	Self-reported bullying had an average Cronbach’s alpha of 0.84 at T1 and T2 and a correlation of 0.45. Peer-reported bullying had an average Cronbach’s alpha of 0.895 with a correlation of 0.72 between T1 and T2.	Self- reports: Comprehensive Scales of Traditional Peer Victimization “CSTPV” (Morrow et al., 2014) Peer-reports: CSTPV (Morrow et al., 2014) with peer nominations	Self-reported victimization had a Cronbach’s alpha of 0.94 and a correlation of 0.61 between T1 and T2. Correlation between T1 and T2 for peer-reported victimization was 0.58.
Green et al. (2020)	New Zealand	2–6 (6–10)	Pre-KiVa Post-KiVa (same group, 1 year later)	1,175 985	Pre-test (prior to any knowledge of KiVa) Post-test (1 year after KiVa’s implementation)	Self-reports: questionnaire (Olweus, 1996)	Significant decrease in the frequency of bullying 1 year after KiVa’s implementation.	Self-reports: questionnaire (Olweus, 1996)	Significant decrease in victimization 1 year after KiVa’s implementation, however results varied by gender and grade level (i.e., bigger impact on victimization rates for females).
Axford et al. (2020)	United Kingdom	2–5 (7–11)	Intervention (KiVa; KiVa also offered for another year post trial) Waitlist control (KiVa offered for 2 years post trial)	1,588 —Intervention 1,892 —Waitlist control	Pre-test (June/July 2013) Post-test (June/July 2014)	Self-reports: questionnaire (Olweus, 1996)	No significant effect of the intervention on self-reported bullying	Self-reports: questionnaire (Olweus, 1996)	No significant effect of the intervention on self-reported victimization
Huitsing et al. (2020)	The Netherlands	3–4 (Dutch grades 5–6) (mean = 8.7)	Intervention (KiVa) Control (no KiVa; “care as usual”)	3,309—Intervention 1,405—Control	First baseline assessment (before summer) Second baseline assessment (beginning of new school year) First follow-up test (end of school year) Second follow-up test (beginning of new school year) Post-test (end of second school year)	Self-reports: questionnaire (10 items) (Olweus, 1996)	Self-reported bullying decreased over time in both conditions. By the 2-year post-test, students in KiVa schools had significantly lower rates of bullying. There were interaction effects of KiVa on bullying at all 3 follow-up assessments in the intervention condition.	Self-reports: questionnaire (10 items) (Olweus, 1996)	Over time, the number of victimized students decreased in both the control and intervention conditions, though this effect was stronger in KiVa schools. At the 1-year follow-up test, the intervention effect was not significant at reducing victimization, though the effect was in the right direction. By the 2-year post-test, the effect on decreasing victimization in the intervention condition as opposed to the control was significant on the maximum score but not the global score.
López-Catalán et al. (2022)	Spain	Primary school (6–12) Secondary school (12–16)	Pre-Kiva Post-Kiva (same group, measured a year after KiVa’s implementation)	3,738	Pre-survey Post-survey (1 year later)	Self-reports: questionnaire (Olweus, 1996)	Following the first year of KiVa implementation, there was a 9.3% increase in students who claimed they did not bully other students and decreases in the percentages of students who claimed they bullied others. A Chi-square test confirmed that statistically less children were perpetrators of bullying 1 year following KiVa’s implementation.	Self-reports: questionnaire (Olweus, 1996)	Following the first year of KiVa implementation, there was an 8.4% increase in students who claimed they were not victims of bullying. Following the first year of KiVa implementation, there was a 2.5 and 5.8% decrease in students who claimed they were bullied once or twice a month and bullied frequently, respectively. A Chi-square test confirmed that statistically less children were victims of bullying 1 year following KiVa’s implementation.
Valenzuela et al. (2022)	Chile	5–6 (10–12)	Full KiVa (includes computer game) Partial KiVa (does not include computer game) Non-treatment Control (No KiVa)	1,363 —Full KiVa 1,260 —Partial KiVa 1,345 —Control	Pre-test (beginning of school year) Post-test (end of school year)	Self-reports: questionnaire—additional item for witnessing bullying added —Revised Olweus Bully/Victim Questionnaire (Gaete et al., 2021)	Significant reduction in witnessing bullying in the partial KiVa group as compared to the control group (stronger effect in 5th graders). No significant difference between partial KiVa condition and control for self-reported bullying. No significant effect of full KiVa intervention as opposed to control or full KiVa as opposed to partial on self-reported bullying and witnessing bullying.	Self-reports: questionnaire—Revised Olweus Bully/Victim Questionnaire (Gaete et al., 2021)	Victimization was significantly lower in the partial KiVa group than in the control group (stronger effect in 5th graders). No significant effect of full KiVa intervention as opposed to control or full KiVa as opposed to partial on self-reported victimization.

The cutoff point for statistical significance is *p* < 0.05.

The table shows that, in seven out of the eight studies, KiVa had a statistically significant positive effect on reducing both victimization and bullying on self-report measures (all studies) and peer-report measures (2 studies). The main findings of these studies, as outlined in the table, and they highlight that KiVa is effective and can be implemented outside of Finland. This bolsters the support for KiVa as an anti-bullying intervention, as it confirms that the effectiveness of KiVa is not bound to one setting or culture, but rather that it is an intervention that can be universally implemented to address school bullying. Moreover, when determining the effectiveness of any psychosocial intervention, it is important for similar or replication studies to be conducted by scholars who were not creators of the intervention. Of the XX studies conducted outside of Finland, YY did not involve researchers involved in the development of KiVa. Despite these positive results, it is also important to consider that the results of two of these studies, Huitsing et al. (2020) and Van der Ploeg et al. (2016), demonstrate that KiVa’s effectiveness appears to dwindle over time, consistent with the results of some of the original Finnish studies. The reason for this is unclear and future research must be done to determine why this occurs and how maintain KiVa’s effectiveness over time. We theorize that adding “booster” sessions of KiVa could be beneficial.

## 3 Conclusion

Thus, with regard to KiVa’s general status as a preventive intervention to reduce school bullying as well as improve bystander responses to bullying, the data suggests that KiVa is an effective intervention technique. Most of the research regarding KiVa’s effectiveness specifically at changing bystander behavior has taken place in Finland, and more research is needed in countries other than Finland, to ensure that KiVa’s ability to improve bystander behavior is not limited to one cultural context. Though the data from the Netherlands and even Chile on the general effectiveness of the program suggest that KiVa is effective at improving bystander responses to bullying cross-culturally, further studies are still needed. Despite the very impressive record of success, the crowning achievement remains for future studies: showing that KiVa not only leads to a reduction in bullying and an improvement in bystander behavior, but also, by including a mediation analysis shows that it is the improvement of bystander behavior rather than some other feature of the intervention that accounts for the reduction in bullying.

### 3.1 New directions in intervention: a more active role for bystanders

The possible benefits of moving well beyond preventive programming to more intensive peer-mediated school interventions are just beginning to be explored. These interventions are inspired by generations of research on peer-mediated interventions for children with special learning needs, very often conditions on the autism spectrum. The interventions are administered by classmates who show leadership potential under the guidance of teachers and/or mental health professionals (Strain and Bovey, 2015).

The very limited results to date come from two studies conducted in Spain, with mixed results. Pina et al. (2021) developed the “Count on Me” program. Pupils elected the student leaders whom they wanted to serve as peer mediators among the students who volunteered. The teachers helped the volunteer leaders learn how to recognize, point out, and praise positive behaviors by both teachers and pupils. Based on specific rules and guidelines, the mediators learned how to resolve conflicts. The peer mediation intervention continued for several months. The experimental group that participated in the peer mediation program experienced reduced violence and increased prosocial behavior in comparison with the control group. The effects of the Count on Me program on bystander behavior have yet to be studied. It is possible to conceive of this type of program as guided practice in becoming a prosocial bystander. However, another study in Spain on a peer-mediation program failed to yield a significant improvement in pro-victim attitudes, bystander roles, or school climate (Villanueva Badenes et al., 2022).

Pending further study, there is no strong reason to believe that these more intrusive peer-mediated interventions improve bystander behavior more than the better-established KiVa program. However, these two small studies on peer mediation, with conflicting results, cannot be considered conclusive. Future studies should seek to focus on which specific components and mechanisms of KiVa lead to positive change in situations of school bullying. As well, the null results produced by some studies of KiVa should be investigated. It is important to note that the studies available to review establish the effectiveness of KiVa on bullying and victimization. These studies do not necessarily indicate that it is the bystander focus that accounts for the outcomes reported or that KiVa is the only possible intervention that could be effective; these issues would have to be explored in new studies in which these issues are explored systematically as different intervention conditions.

## Footnotes

^1^ What is KiVa? KiVa Program & University of Turku. Available at: https://www.kivaprogram.net/what-is-kiva/   (Accessed September 20, 2024).
